# Genetic diversity of the highly variable V1 region interferes with Human Immunodeficiency Virus type 1 envelope functionality

**DOI:** 10.1186/1742-4690-10-114

**Published:** 2013-10-24

**Authors:** Meriem Hamoudi, Etienne Simon-Loriere, Romain Gasser, Matteo Negroni

**Affiliations:** 1 Architecture et Réactivité de l'ARN, CNRS, IBMC, Université de Strasbourg, 15 rue René Descartes, 67084 Strasbourg, Cedex, France; 2 Present address: Institut Pasteur, Unité de Génétique Fonctionnelle des Maladies Infectieuses, 28 rue du Docteur Roux, 75724 Paris CEDEX 15, France; Centre National de la Recherche Scientifique, URA, CNRS3012 Paris, France

**Keywords:** HIV-1, Envelope, gp120, Variable regions, Viral entry

## Abstract

**Background:**

The HIV envelope (Env) promotes viral entry in the host cell. During this process, Env undergoes several conformational changes to ensure its function. At the same time, the gp120 component of Env is the protein of the virus presenting the largest genetic diversity. Understanding how the virus maintains the balance between the competing requirements for maintenance of functionality and antigenic variation of this protein is central for the comprehension of its strategies of evolution and can highlight vulnerable aspects of its replication cycle. We focused on the variable domains V1 and V2 of the HIV-1 gp120 that are involved in conformational changes and are critical for viral escape from antibody neutralization.

**Results:**

Despite the extensive sequence diversity found in the epidemic for these regions and their location on the external face of the protein, we observed that replacing V1V2 of one primary isolate with that of another severely interferes with Env functionality in more than half of the cases studied. Similar results were obtained for intra- and intersubtype chimeras. These observations are indicative of an interference of genetic diversity in these regions with Env functionality. Therefore, despite the extensive sequence diversity that characterizes these regions in the epidemic, our results show that functional constraints seem to limit their genetic variation. Defects in the V1V2 chimeras were not relieved by the insertion of the V3 region from the same isolate, suggesting that the decrease in functionality is not due to perturbation of potential coevolution networks between V1V2 and V3. Within the V1V2 domain, the sequence of the hypervariable loop of the V1 domain seems to be crucial for the functionality of the protein.

**Conclusions:**

Besides the well-documented role of V1V2 in the interplay with the immune response, this work shows that V1 is also involved in the selection of functional envelopes. By documenting a compromise between the opposing forces of sequence diversification and retention of functionality, these observations improve our understanding of the evolutionary trajectories of the HIV-1 envelope gene.

## Background

Genetic diversification and natural selection are the essential processes that prompt adaptation to the environment. Living under the constant pressure imposed by the immune system constrains viruses, and in particular those producing persistent infections. These constraints involve perpetual change of their epitopes while preserving the functionality of the individual genes and gene networks. The human immunodeficiency virus type I (HIV-1) constitutes one of the most well-characterized examples of genetic variation, with an impressive sequence diversity documented for this virus in the AIDS pandemic [1]. The extent of such genetic diversity in HIV-1 implies that the viral components are highly tolerant of sequence variation. The HIV-1 component presenting the highest degree of sequence diversity is the extra-viral part of the envelope protein, comprising the glycoprotein gp120. The HIV-1 envelope enables viral entry into the target cell, a process mediated by recognition of the CD4 receptor and CCR5 or CXCR4 coreceptors at the surface of the cell membrane [2-9]. HIV-1 Env is composed of glycoproteins gp41 and gp120, which form a non-covalent complex [10]. The transmembrane gp41 keeps the Env machinery associated with the viral membrane, whereas the surface gp120 is located completely on the outside face of the viral lipid bilayer. Trimers of this heterodimer constitute the functional form that mediates viral entry [10]. After binding to the CD4 molecule present at the surface of target cells [4,9], gp120 undergoes conformational changes that expose a cryptic binding site for the coreceptor molecule [5-7,11]. The variable regions V1, V2, and V3 have all been implicated in the conformational changes required to expose the coreceptor binding site [12-15]. Upon interaction with the coreceptor, further conformational changes occur, leading to the exposure of the fusion peptide with the gp41 ectodomain and the transition of gp41 into a pre-hairpin intermediate [16-19]. These changes will then promote the fusion of the viral and target cell membranes [20-22]. The need for these conformational transitions requires a remarkable flexibility of the protein and its functional domains that are formed in the ternary and, possibly, the quaternary structure of the protein [23,24].

An ideal means to conciliate the opposing requirements of antigenic variation and the preservation of functionality of a protein requires the complete functional separation of its domains. This can ensure the achievement of these two goals through their structural independence. Thus, the regions that are more accessible to the immune system are free to undergo extensive sequence diversification to counteract the immune response. On the other hand, the regions that are responsible for enzymatic reactions or structural interactions could remain essentially unvaried, preserving their functionality. gp120 is evocative of such a task specialization. This protein is organized in five constant regions (C) that alternate with five variable regions (V) [25,26]. This organization partially reflects the spatial arrangement of the protein, in that the V regions are located mostly on the external part of the protein, whereas the C regions are globally located more in the internal portion [27,28]. The location of the V regions in the external part of the protein [27-29] and their extensive genetic diversity [1] imply that one of their functions is to serve as a decoy for the immune response, whereas the more conserved internal core provides the scaffold that stabilizes the structure of the protein [30,31]. In parallel with this architectural organization, the virus has developed various other strategies to escape the immune response raised by the host. Among these strategies is the masking of the coreceptor binding site through the presence of the variable loops until the virus has docked onto the target cell. The presence of multiple N-linked glycosylation sites in gp120 [27,29,32,33], and particularly in its variable regions, constitutes another means of restricting recognition of the protein by the antibodies raised by the host. In addition, the variable domains evolve in natural infections, with changes in size, sequence, and glycosylation pattern occurring during disease progression, thus increasing neutralization resistance [9,18,32,34-37]. The variable domains have also been shown to be involved in some essential processes for Env functionality, such as the formation of the coreceptor binding site [32,38-42] or the role of V3 in the determination of the viral tropism [39]. Despite their high genetic variability, conserved structural features of these regions are also beginning to emerge, suggesting that the V regions may play other important roles in the functionality of gp120 [43]. These conserved structural motifs essentially concern the V1V2 and V3 regions. Indeed, V3 is considered as the most conserved of the variable regions and presents conserved base and tip of the loop with variable intervening regions [44]. For the V1V2 region, an organization in four conserved β-sheets with two intervening variable loops has been described [31].

V1 and V2 are the most variable regions in gp120, and their predominant involvement in the development of neutralizing antibodies has been described in several studies, particularly after the development of the RV144 vaccine trial, which elicited antibodies against this region [45-47]. Studies on the functional role of these regions were generally conducted with deletion or mutation of these regions. It was shown that mutants with either V1, V2, or both deleted loops did not abrogate the capacity of the virus to replicate in tissue culture, even if these mutants were less efficient than their wild-type counterparts [15,48-53]. Here, through the study of chimeras in which the variable regions V1V2 were replaced using primary isolates from different subtypes, we addressed the issue of the importance of these regions, in particular the poorly studied V1 loop, in the maintenance of the functionality of the envelope protein.

## Results

### The replacement of the homologous structural V1V2 region of gp120 between primary isolates affects Env functionality

**Figure 1:**
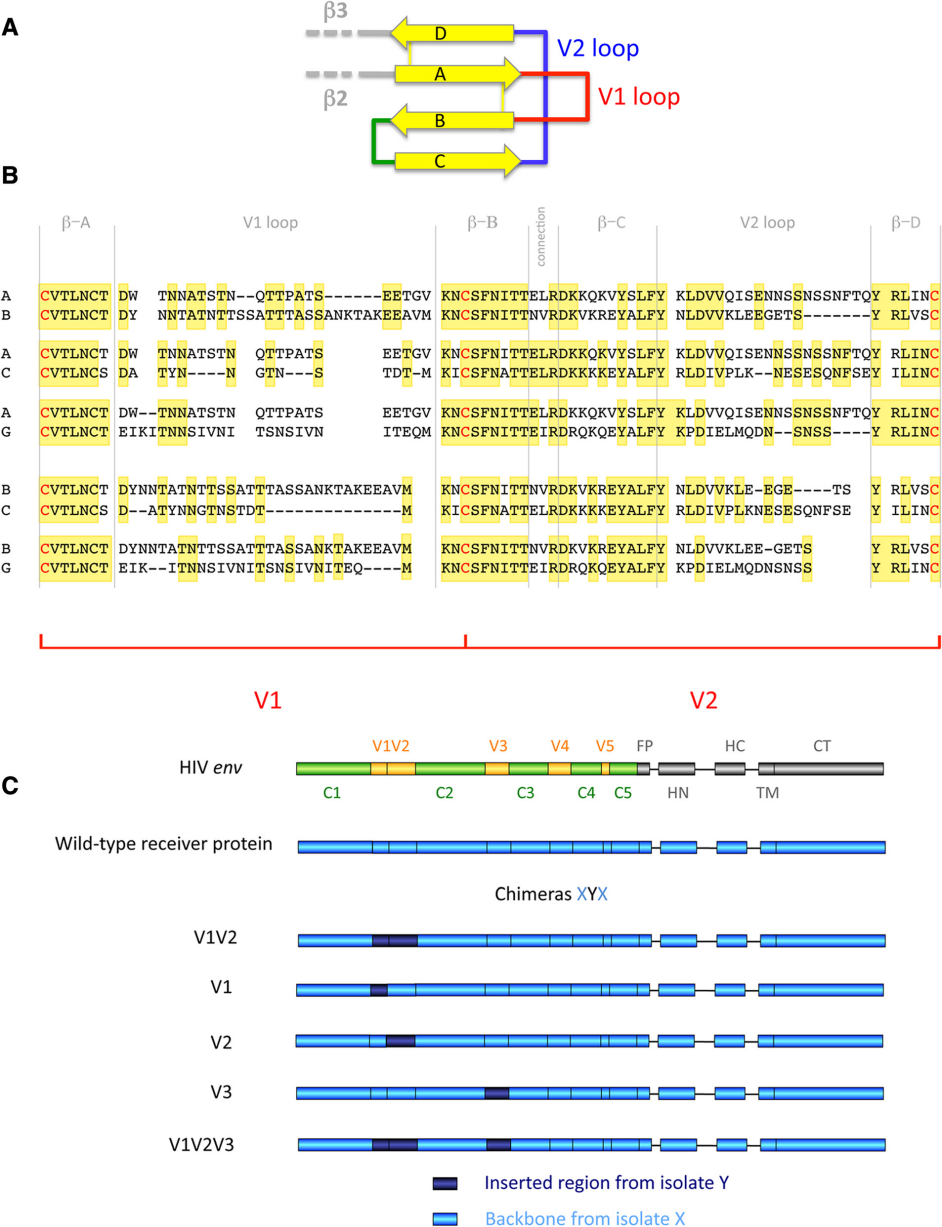
**Strategy and sequences used in the present study.***Panel ****A****. Representation of the V1V2 region according to the structure of McLellan* et al. [31]*.* The four β-sheets are indicated by yellow arrows, the unstructured loops V1 and V2 are indicated in red and blue, respectively, whereas the small loop connecting β-sheets B and C is drawn in green. The start of the β-sheet B differed between the two isolates used in McLellan et al. (CAP 45 and ZM109). Here, we considered the minimal size of the β-sheet B, which is the one from the isolate CAP 45. *Panel ****B***. *Amino acid sequence alignment of the V1V2 region for the isolates used to generate the main five chimeras characterized in this study.* Identical residues are in a yellow background, and hyphenations indicate gaps in the alignment. The C residues that define the borders of the V1 and V2 regions are indicated in red. The delimitations of the V1 and V2 regions is given, as a reference, at the bottom of the drawing. The vertical gray bars define the borders of the structural domains defined by McLellan and colleagues [31]. The four β-sheets are indicated as βA, B, C and D, while the region indicated as "connection" corresponds to the mini loop connecting β-sheets B and C (see text for details). Alignments were performed using MUSCLE 3.8 [55]. *Panel ****C***. *Schematic representation of the chimeric HIV-1 envelopes used in this study.* The structure of the HIV-1 envelope gene is given at the top as a reference, with the constant and variable regions of the gp120 region in green and yellow, respectively. The fusion peptide (FP), helix N (HN), helix C (HC), transmembrane domain (TM) and cytoplasmic tail (CT) of the gp41 are indicated in gray.

In a previous work, we showed that when recombination between primary isolates of HIV-1 group M led to a replacement of a region that included the V1V2 portion of gp120, recombinant envelopes displayed a marked decrease in functionality [54]. To better understand this observation and investigate the impact of genetic variation in the V1V2 region on the functionality of the HIV-1 envelope, chimeric envelopes were generated by replacing the V1V2 region of one primary isolate with the corresponding region of other primary isolates. The V1V2 region is organized in four β-sheets and two loops as indicated in Figure 1A [31]. The β-sheets are more fairly conserved than the loop portions, as indicated in Table 1 and Additional file 1: Table S1. In this work we will refer to V1V2 as a "region", to V1 or V2 as a "domain", and to the hypervariable loops as "loops".

**Table 1:** Amino acid sequence identity of the structural components of the V1V2 region for the chimeras

**chimeras**	**V1**	**V2**	**β-A**	**V1 loop**	**β-B**	**connect**	**β-C**	**V2 loop**	**β-D**
A/B	52.5	50.0	100.0	36.7	100.0	33.3	54.5	38.5	60.0
A/C	46.9	63.0	85.7	31.8	77.8	100.0	72.7	42.9	80.0
A/G	38.2	56.5	100.0	12.5	100.0	66.7	45.5	38.1	100.0
B/C	37.5	54.5	85.7	23.3	77.8	33.3	81.8	33.3	40.0
B/G	42.5	52.5	100.0	23.3	100.0	33.3	72.7	20.0	60.0

Results are based on the structure described in reference [31]. The values of sequence identity are given in percentage. The individual regions are defined as in Figure 1A. The similarity table based on the Blosum62 matrix is provided in Additional file 1: (Table S1).

We focused on the study of chimeras derived from isolates of HIV-1 belonging to the phylogenetic group responsible for the vast majority of the infections, group M. This group is further subdivided in various subtypes (A-D, F-H, J, and K) [56]. Primary isolates belonging to subtypes A, B, C, and G were employed in the present study. These subtypes were frequently found to produce intersubtype recombinant forms in the epidemics [57]. We initially chose isolates that we previously characterized in the study on genetic recombination mentioned above [54,58-60]. The sequences of the V1V2 region of these isolates are shown in Figure 1B. As an example, we refer to the isolates as "isolate A", "isolate A1"… for isolates that belong to subtype A of group M, as "isolate B" for the isolate from subtype B used, and so on. This nomenclature is not intended to generalize the behavior of a given isolate to all the isolates of the same subtype.

The chimeric envelopes used in this study are referred to as XYX, where X indicates the isolate providing the backbone (called the receiver) and Y indicates the isolate providing the insert, called the donor (Figure 1C). As an example, the chimera AGA V1V2 is comprised of the envelope of an isolate from subtype A carrying a V1V2 region from an isolate of subtype G (see Methods for details on the sequences used throughout the study).

The functionality of the chimeric envelopes was defined by their ability to mediate viral entry into target cells. pNL4.3-Env^-^-Luc^+^ virions complemented by the individual chimeric envelopes are used for this test, as described in Methods. To understand whether the replacement of the V1V2 region altered the functionality of the receiver protein, the functionality of each chimera was compared with that of the corresponding wild-type receiver protein (a comparison of the functionality of the chimeras with respect to the wild-type donor protein is also provided, in Additional file 2: Table S2).

**Figure 2:**
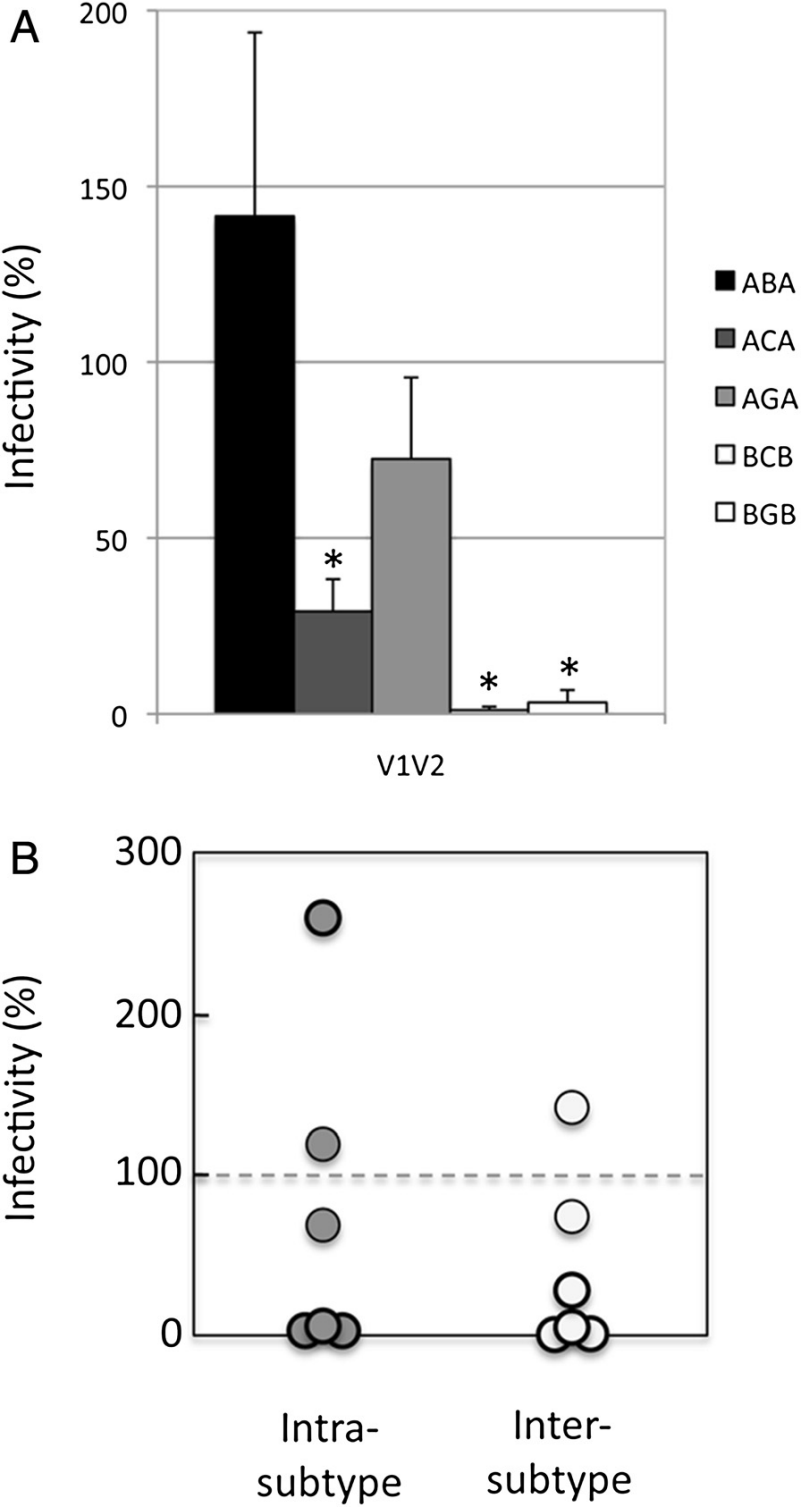
**Functionality of the V1V2 chimeric envelopes in viral entry tests.***Panel ****A***. *Functionality of the V1V2 chimeras.* The level of functionality of the V1V2 chimeras in viral entry tests for the main 5 chimeras characterized in this study is given relative to the functionality of the wild-type receiver protein (Env from the isolate of subtype A for the ABA, ACA, and AGA chimeras, and from subtype B for BCB and BGB chimeras). Values are the average of 4 to 5 independent experiments. The asterisk indicates a significant difference (p < 0.001) in the functionality of the chimera with respect to the corresponding wild-type receiver protein. *Panel ****B****. Functionality of V1V2 intra- and intersubtype chimeras.* The functionality of six intrasubtype (gray circles) and six intersubtype (white circles) chimeras is given relative to the functionality of the corresponding wild-type receiver Env. Thicker circles indicate significant differences (p < 0.001) in the functionality of the chimera with respect to the corresponding wild-type receiver protein. Values are the average of 3 to 5 independent experiments. Details regarding the sequences used for this part of the study are given in the Methods section.

A significant decrease in viral entry was observed in three of the cases studied, with a drop in functionality to less than 30% for the ACA chimeras and to almost undetectable levels for the BCB and BGB chimeras, as indicated in Figure 2A. For the AGA chimeras, only a modest decrease was observed (functionality higher than 70% of the corresponding wild-type receiver protein; p = 0.10), whereas the ABA chimera displayed a slight (1.4 fold; p = 0.27) increase in functionality with respect to the wild-type A backbone protein. Differences in the level of functionality cannot be accounted for by differences in the levels of expression of the chimeras as shown by Western blot (Additional file 3: Figure S1).

Given the overall extensive genetic diversity present in V1V2 in the viral population and their location on the external face of the protein, it was expected that these regions could be easily exchanged. The strong decrease in functionality observed in three of the five V1V2 chimeras was, therefore, unexpected.

To extend these observations to the case of intrasubtype chimeras, we constructed six intrasubtype V1V2 chimeras (two of subtype A, three of subtype C, and one of subtype G; see Methods). To generate comparable datasets for intra- and intersubtype chimeras, we also generated an additional intersubtype chimera (between isolates from subtype B and C; see Methods). The amino acid sequence identity ranged from 45.7 to 56.4% among the intersubtype chimeras and from 50.0 to 66.7% among the intrasubtype chimeras (Table 2). Seven of the twelve chimeras displayed significantly decreased functionality (p < 0.005) with respect to the corresponding wild-type proteins (4 inter- and 3 intrasubtype chimeras), with a drop in functionality for 6 of the chimeras below 5% of that of the corresponding wild-type receiver Env, while the seventh showed a residual functionality of 29% (Figure 2B, and Table 2). The remaining chimeras exhibited levels of functionality that were in the range of those of the corresponding wild-type receiver proteins, except for the intrasubtype chimera GG1G, which displayed a 2.4-fold increase. The average level of functionality of the intersubtype chimeras (41 ± 56%) was lower than that of the intrasubtype chimeras (73 ± 95%). However, given the large range of the values observed within intra- as well as intersubtype chimeras, the difference between the two datasets was not significant (p = 0.57). The decrease in functionality that we originally observed by replacing V1V2 between intersubtype isolates is therefore also found when considering genetically closer isolates.

**Table 2:** Sequence identity and entry efficiency of V1V2 inter- and intrasubtypes chimeras

		**Sequence identity (%)**	**Viral entry (%)**	**SD (%)**
Intersubtype	A/B	51.2	141.5	52.2
	A/C	56.4	29.1	9.2
	A/G	48.8	72.5	23.0
	B/C	46.4	1.1	1.0
	B/C3	45.7	0.5	0.3
	B/G	47.5	3.2	3.6
Intrasubtype	A/A1	66.7	69.4	38.6
	A/A2	62.8	115.9	20.1
	C/C1	60.0	1.2	0.6
	C/C2	50.0	2.8	1.5
	C/C3	57.5	0.9	0.6
	G/G1	54.1	257.1	27.4

In all cases, the values are given relative to those observed for the corresponding wild-type receiver protein. The results for viral entry represent the mean values derived from 3 to 5 independent experiments. SD, standard deviation.

### The variable domain V3 does not influence the functionality of the V1V2 chimeras

**Figure 3:**
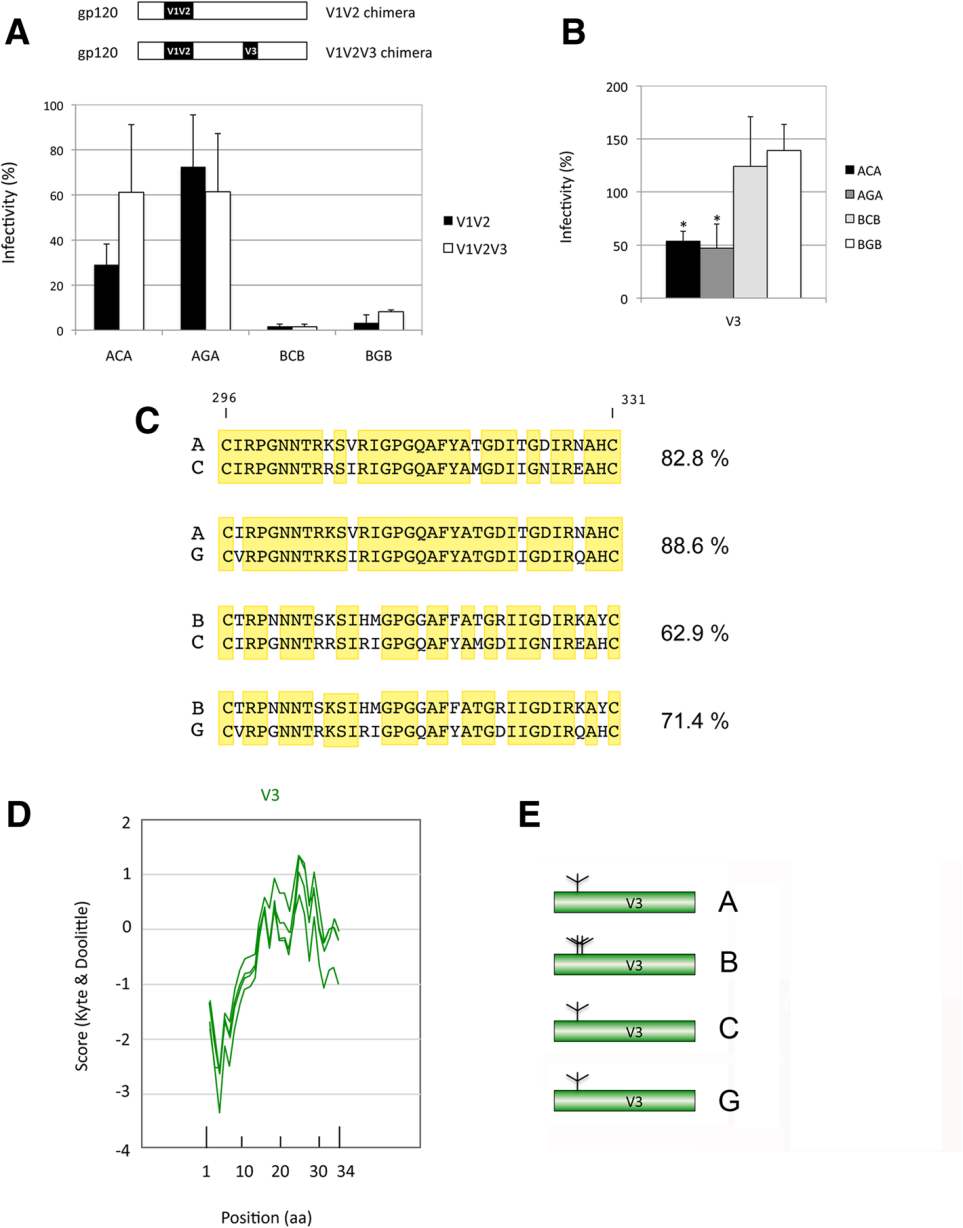
**Effect of the presence of homologous V1V2 and V3 regions on the functionality of the chimeric envelopes.***Panel ****A****. Comparison of the functionality of V1V2/V3 chimeras with respect to V1V2 chimeras.* The values for the V1V2 chimeras (from Figure 2A) are given as a reference in black. *Panel ****B****. Functionality of V3 chimeras.* Values are the average of 4 to 5 independent experiments in both panels. The asterisk indicates a significant difference (p < 0.001) in the functionality of the chimera with respect to the corresponding wild-type receiver protein. *Panel ****C****. Amino acid sequence alignment of the V3 region for the isolates used to generate the chimeras characterized in Panel ****B****.* Identical residues are in a yellow background. The sequences are shown including the C residues that border the V3 region. Alignments were performed using MUSCLE 3.8 [55]. The percentage of identity between the isolates is given on the right of each alignment. *Panel ****D****. Hydrophobicity profile of the variable region V3.* The profile of the four sequences most intensively studied here (isolates A, B, C, and G) is given. The position in amino acids is given on the x axis, with numbering starting from the first amino acid (N-ter) after the cysteine residue that marks the border of the C2-V3 transition. *Panel ****E****. Potential N-Glycosylation sites of the variable region V3 for each subtype used.* The location of N-glycosylation sites is indicated. Only sites with a predicted N-glycosylation potential >0.5 according to Server NetNGlyc 1.0 [64] are shown.

Several lines of evidence suggest that V1V2 and V3 are in close spatial proximity and that this proximity is involved in epitope masking from the immune response [61-63]. To understand whether the decrease in functionality observed for the V1V2 chimeras was due to the phylogenetic discordance between V1V2 and V3, we inserted in the V1V2 chimeras the V3 domain from the same isolate. In this case, a recovery in functionality would suggest that V1V2 had coevolved with V3. For this analysis, we focused on the ACA, AGA, BCB, and BGB V1V2 chimeras that, as shown in Figure 2A, exhibited a decreased functionality compared to the wild-type receiver protein. To this end, we tested the ability to mediate viral entry into target cells of chimeras where the V1V2 and V3 sequences came from the same isolate (V1V2/V3 chimeras; see Figure 1C). Apart from the ACA chimera, for which a twofold (although not significant; p = 0.09) increase in functionality was observed, in none of the cases the insertion of the homologous V3 region in the V1V2 chimeras restored the functionality of the simple V1V2 chimeras (p > 0.05) (Figure 3A). All of the V1V2/V3 chimeras remained at levels of functionality significantly lower (p < 0.05) than the corresponding wild-type receiver proteins. Therefore, the alteration of the functionality observed in the V1V2 chimeras does not seem to be due to the presence of a V3 domain from a different phylogenetic origin.

The observation that swapping of the V3 domain only marginally affects the functionality of Env is also confirmed by the replacement of V3 alone (V3 chimeras, Figure 1C). With V3 chimeras, the decrease in functionality of the envelopes was less marked than for the V1V2 chimeras. Indeed, no decrease was observed for two cases, and for the other two, the decrease was limited to 50% (Figure 3B). The level of expression of the different chimeras cannot account for the differences in functionality observed (Additional file 3: Figure S1, supplementary material).

The higher level of sequence identity of the V3 sequence (Figure 3C), which ranged from 62.9% to 88.6% for the isolates used, the similarity of hydrophobicity profiles and predicted glycosylation patterns (Figure 3D and E) could explain the good tolerance to swapping this domain between isolates.

### Relative contribution of V1 and V2 domains to the functionality of the chimeras

**Figure 4:**
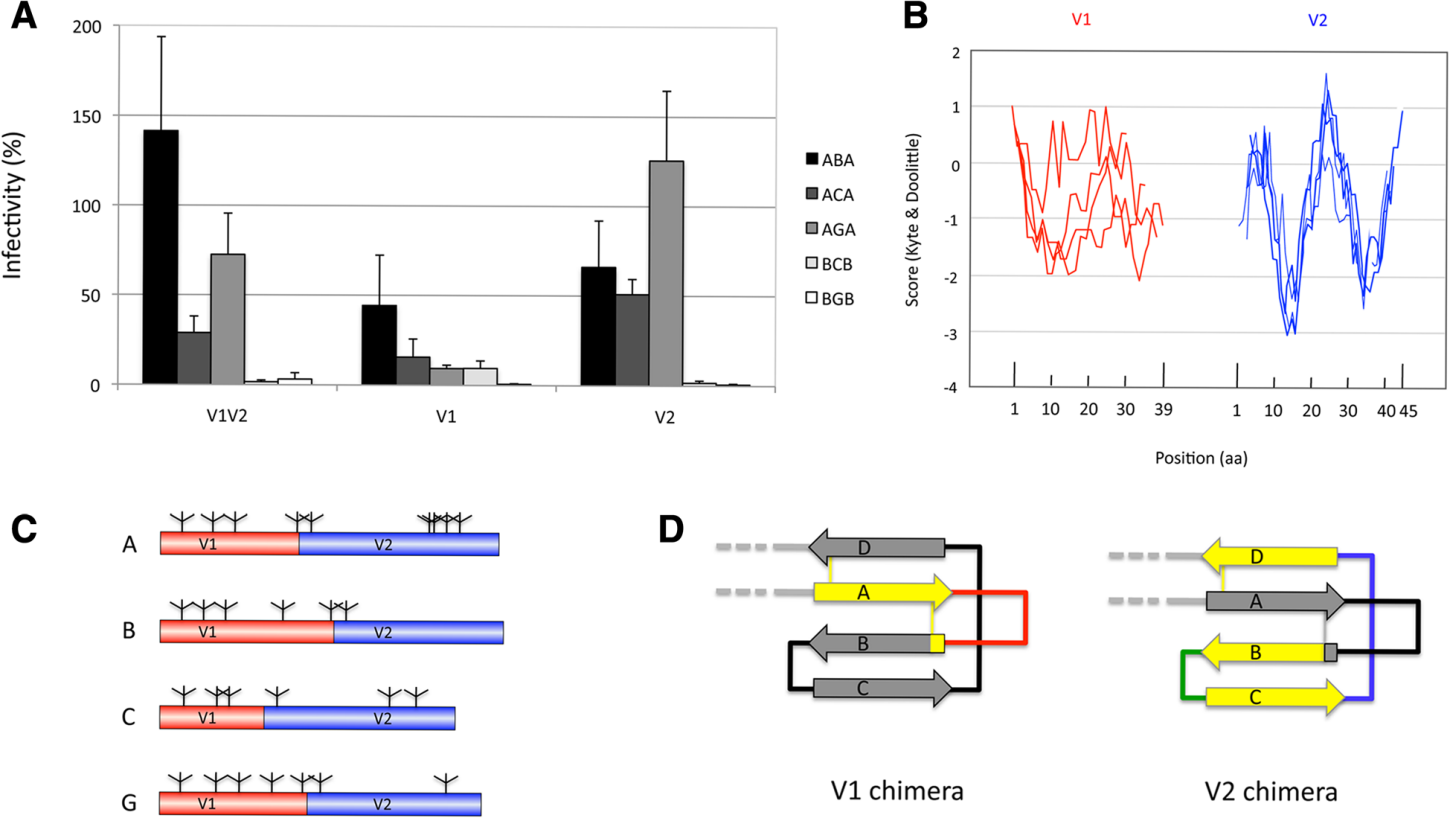
**Implication of variable domains V1 and V2 in the loss of functionality of the chimeras.***Panel ****A****. Functionality of V1 and V2 chimeras.* The values for the V1V2 chimeras (from Figure 2A) are given as a reference for comparison with the V1 and V2 chimeras. The values are the average of 3 to 6 independent experiments. In all cases, the values are given relative to those observed for the corresponding wild-type receiver protein. *Panel ****B****. Hydrophobicity profile of the variable regions V1 and V2.* The profile of the four sequences most intensively studied here (isolates A, B, C, and G) is given for V1 in red and for V2 in blue. The position in amino acids is given on the x axis, with numbering starting from the first amino acid (N-terminal) after the cysteine residue that marks the border of the C1-V1 transition for V1 and the V1-V2 transition for V2. *Panel ****C****. Potential N-Glycosylation sites of the variable regions V1 and V2 for each subtype used.* The locations of the N-glycosylation sites with an N-glycosylation potential >0.5, as predicted according to the Server NetNGlyc 1.0 [64] in V1 or V2 are indicated in the red and blue regions, respectively. *Panel ****D****. Representation of the V1V2 region in the V1 or in the V2 chimeras according to the structure of McLellan* et al. [31]*.* The representation is as in Figure 1A. The colored part of the drawing corresponds to the parts that were replaced.

The V1V2 region is composed of two distinct domains, V1 and V2 [31] that are bordered by a disulfide bond. To evaluate the contribution of each of these two domains to the observed decrease in functionality of the V1V2 chimeric Env, we constructed chimeric Env proteins carrying envelopes where only the V1 or the V2 domain was replaced (V1 and V2 chimeras, Figure 1C). We focused on the five chimeras presented in Figure 2A. All the V1 chimeras displayed a decreased functionality with respect to the corresponding wild-type proteins (Figure 4A). With the exception of the ABA chimera (level of functionality of 44%), the residual functionality was never above 15%. Concerning the V2 chimeras, the perturbation of the functionality was globally less dramatic. Indeed, the functionality of the AGA chimera did not differ significantly from that of the wild-type backbone A protein, whereas for the ABA and ACA chimeras a significant (p < 0.05) reduction was observed, albeit only by around a factor of two (Figure 4A). For BCB and BGB chimeras, instead, swapping of V2 reduced the functionality to levels never above 3%. The overall more limited perturbation of the activity observed with the V2 chimeras can be accounted for by the lower divergence of hydrophobicity profiles and predicted glycosylation patterns observed in this region with respect to V1, for which important differences were observed among the isolates (Figures 4B and 4C). Also in this case, the level of expression of the various chimeras could not account for the differences in functionality observed (Additional file 3: Figure S1, supplementary materials).

These results were then analyzed based on the crystal structure of these domains, which was recently solved by McLellan and colleagues [31]. The V1V2 region in complex with the neutralizing antibody PG9 are composed of 4 stranded anti-parallel β-sheet domains (Figure 1A). According to that structure, the replacement of V1 from cysteine to cysteine (126–157, HXB2 numbering) as we have implemented in our chimeras, leads to the replacement of β-sheet A, the V1 loop, and the beginning of β-sheet B (Figure 4D). In the ABA, AGA, and BGB chimeras this results in changes restricted to the V1 loop (Figure 1B), indicating that this loop is the one responsible for the drop in functionality observed in these chimeras. For the other two V1 chimeras (ACA and BCB), in addition to the V1 loop, one substitution (S vs T) was also present in β-sheet A and one (I vs N) in the beginning of β-sheet B (Figure 1B). Therefore, in these cases, these substitutions may have contributed to the decreased functionality observed in these two chimeras. In the case of the V2 chimeras, the most extensive sequence variation was due to the V2 loop even if, in this case, more differences were observed also elsewhere, particularly in β-sheet C (Figure 1B and Table 1).

### Membrane fusion ability of the chimeric envelopes

**Figure 5:**
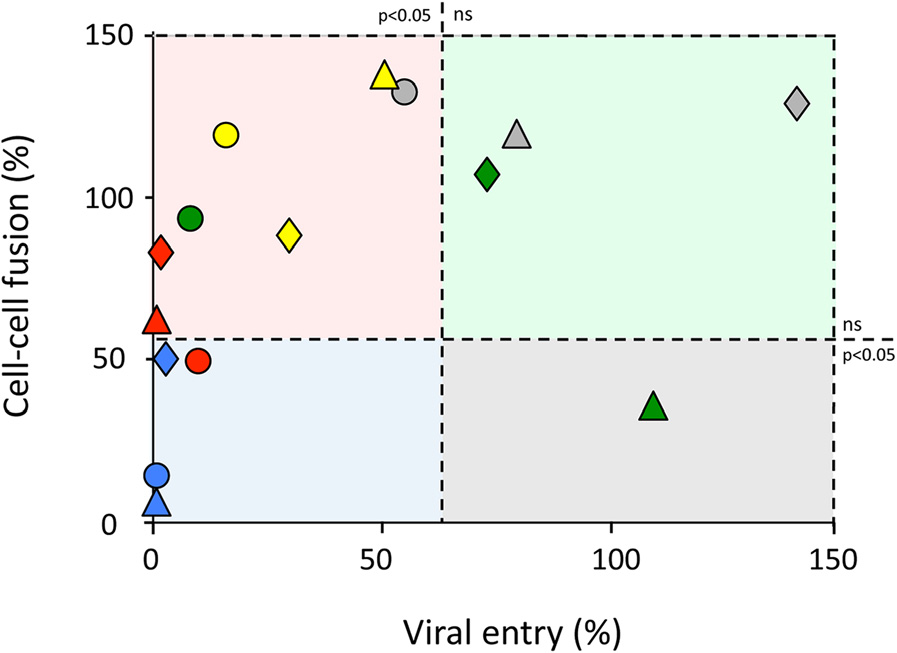
**Correlation between cell-cell fusion and viral entry for envelopes carrying an exogenous V1, V2, or V1V2 region.** Values are the average of 3 to 5 independent experiments for viral entry assays or the average of 3 independent experiments for cell-cell fusion assays. The graph is divided into four sections according to whether the proteins located in one quarter displayed or did not display a level of functionality significantly lower than that of the corresponding wild-type protein in viral entry or in cell-cell fusion assays. The threshold of significance is set at p < 0.05, as indicated in the figure (ns = not significant). The bars indicating the threshold of significance are drawn in the median position between the position of the last sample that has a significant decrease in functionality and the first one that does not display a significant difference (gray circle and green diamonds for cell entry; blue diamonds and red triangle for cell-cell fusion). V1V2 chimeras, diamonds; V1 chimeras, circles; V2 chimeras, triangles. Chimeras: ABA, gray, ACA, yellow; AGA, green; BCB, red; BGB, blue.

To obtain insights into the nature of the defect in functionality observed in the envelopes carrying an exogenous V1V2, V1, or V2 region, the chimeras used thus far in the present study were characterized in cell-cell fusion assays. To this end, we used cells expressing the chimeric envelope (producer cells, HeLa-Tat) and CD4+/CCR5+ TZMbl cells (target cells). This assay, which has been previously described [65], consists in measuring the extent of membrane fusion between producer and target cells through the ability of the chimeras to form syncytia upon co-cultivation of these cells. The formation of syncytia leads to the activation of the luciferase reporter gene present in the target cells (see Methods). The ability to mediate cell-cell fusion is given as a percentage relative to the wild-type receiver protein. Envelopes positive for this test can thereby recognize the receptor and coreceptor molecules as well as carry out membrane fusion. The results obtained with this test are given in Table 3 and are plotted in Figure 5 as a function of the efficiency of viral entry.

**Table 3:** Cell-cell fusion assay values of V1V2, V1, and V2 chimeras

		**Cell-cell fusion (%)**	**SD (%)**
V1V2	ABA	131.5	59.9
	ACA	90.4	39.7
	AGA	108.0	18.1
	BCB	84.6	16.0
	BGB	50.7	18.0
V1	ABA	133.0	51.6
	ACA	120.1	52.9
	AGA	93.5	28.1
	BCB	49.8	15.1
	BGB	14.6	8.2
V2	ABA	120.5	29.3
	ACA	138.3	56.6
	AGA	35.1	12.7
	BCB	61.8	17.1
	BGB	4.8	6.4

In all cases, the values are given relative to those observed for the corresponding wild-type receiver protein. The results represent the mean values derived from at least three independent experiments. SD, standard deviation.

The graph in Figure 5 has been divided into four parts defined by whether the difference between the activity of the chimeras and that of the wild-type backbone was significantly different or not, in the viral entry and cell-cell fusion tests (horizontal and vertical bars, respectively). Chimeras falling in the blue section of the graph were significantly less functional than the wild-type backbone proteins in both entry and cell-cell fusion tests (p < 0.05), which is therefore indicative of an intrinsic defect of the proteins. The red section comprises chimeras whose defect in viral entry tests was relieved in the cell-cell entry assay. This result can be indicative of increased shedding of the chimeric gp120 from the viral particle, since such a defect would be compensated in the cell-cell fusion assay by the continuous production of Env molecules in the producer cells. Chimeras in the green section correspond to proteins that retained a level of functionality not significantly different from that of the corresponding wild-type protein in the viral entry assay as well as in the cell-cell fusion test.

Theoretically, no chimeras should fall in the gray section, since envelopes that promote membrane fusion in the context of the viral particle are also supposed to support membrane fusion in the less stringent cell-cell fusion assay. Despite this, the AGA V2 chimera does fall in this region. This observation is explained by the defect, observed by western blot, in the expression of this chimera in the cells used for the cell-cell fusion assay (HeLa-Tat), but not in the viral particles used for the viral entry test, which were produced by HEK 293T cells (see Additional file 3: Figure S1 and Additional file 4: Figure S2). Apart from this case, none of the differences in the levels of functionality of the chimeras in the cell-cell test could be accounted for by their levels of expression (Additional file 4: Figure S2).

## Discussion

Genetic diversity is crucial for HIV escape from the host immune response. As the most external portion of the viral particle, gp120 is the component of the virus most exposed to immune pressure and, consequently, the protein for which genetic diversification likely has the utmost importance. In addition, gp120 is the sole target of neutralizing antibodies thus far identified, and the recent RV144 vaccine trial has shown that the V1V2 of gp120 is the main region involved in the escape from the immune response [45-47]. Although no crystal structure of the entire gp120 protein, including all the variable regions, is available to date, electron tomography, cryoelectron microscopy and analysis of the crystal structures with the variable regions deleted support the view that the variable regions emanate from the central core composed of the constant portions of the protein [43]. The location of these regions on the external portion of the protein and their extensive sequence diversity suggest that they might undergo only minor structural constraints and that they have evolved to maximize their possibility of sequence diversification while preserving the functionality of the protein. Despite these considerations, we observed here that the replacement of the V1V2 region of a given Env of a primary isolate with the V1V2 region of a functional Env from a different primary isolate reduced the functionality to almost undetectable levels in 7/12 cases studied (Figure 2B). With the exception of the G/G1 chimera that displayed an increased functionality (Table 2), the remaining chimeras displayed levels of functionality comparable to those of the corresponding wild-type backbone proteins. Overall, the functionality of Env was perturbed at comparable levels in intra- and intersubtype chimeras. The latter finding underscores the observation that interference with envelope functionality is not limited to the relatively rare case of chimeras involving isolates from different HIV-1 subtypes but has broader validity. Globally, the chimeras tended to have a decreased functionality with respect to the corresponding wild-type proteins, suggesting that, as shown for the case of single amino acid mutants of this gene in previous studies [66-68], chimeric envelopes mostly undergo purifying selection.

Several lines of evidence support a close association between V1V2 and V3 regions. Their implication in the formation of an interacting surface between monomers, or at least their close spatial proximity within the trimer, has been supported by the identification of broadly neutralizing antibodies that recognize an epitope present only in the trimeric form of gp120. This epitope is constituted partially of V1V2 and partially of V3 [63,69]. In addition, V1V2 and V3 jointly protect neutralization-sensitive epitopes that are recognized by cross-neutralizing plasma by creating a shield that has been suggested to result from the juxtaposition of the V1V2 region from one subunit of the Env trimer with the V3 region from another subunit [62]. Relevant to this work, the spatial proximity between V1V2 and V3 raised the issue of a possible coevolution between the V1V2 and V3 regions that could be responsible for the decreased functionality observed for some of our V1V2 chimeras. The results we report here, however, do not support this view because, in the V1V2/V3 chimeras, the simultaneous replacement of V1V2 and V3 from the same isolate did not preserve Env functionality, which remained in the range of what was observed for the simple V1V2 chimeras (p > 0.5 in all cases, Figure 3A). However, the lack of restoration of functionality of Env could also simply reflect the fact that the simultaneous replacement of the V1V2 and V3 regions was not sufficient to obtain their reciprocal proper orientation in the context of the exogenous backbone protein.

In line with the view of limited involvement of V3 in the mechanisms leading to the decreased functionality of the chimeric envelopes studied here, the replacement of V3 alone globally had less dramatic consequences on the functionality of Env compared with that observed for the V1V2 chimeras. The higher level of sequence conservation of V3 [1], which likely reflects the selection for maintaining an optimal conformation of the coreceptor binding site, [43] could explain why the replacement of this domain is better tolerated than that of V1V2. A higher similarity among the V3 than V1V2 domains was also observed at the levels of hydrophobicity, pattern of potential glycosylation sites, and overall size observed between V3 and V1V2 regions (Figure 3, panels D and E).

Several of our V1V2 chimeras presented defects in the viral entry assays that were relieved in the cell-cell fusion test (Figure 5). A possible explanation for such a phenotype is that the chimeras present a decreased stability of the gp120/gp41 complex, defect that would be compensated in the cell-cell fusion assay by the continuous production of Env that migrates at the cell surface. V1 and V2 are in a crucial position for the arrangement of the N- and C-terminal domains, which are involved in the formation of the interface of gp120 contacting the gp41 [70,71]. This situation could potentially influence the stability of the interaction between gp120 and gp41.

In the present study, the replacement of the V1 or V2 domains alone provided important insights for understanding which parts are involved in the perturbation of the functionality of the V1V2 chimeric envelopes. The structure of the V1V2 region in complex with the broadly neutralizing antibody PG9 has been recently solved, revealing an organization in four, fairly conserved, antiparallel β-sheets and two non-structured loops that present the strongest genetic diversity (Figures 1A and 1B and [31]). Even if it is difficult to distinguish the contribution of V1 independently from that of V2, and vice versa, because the replacement of only one domain might trigger an alteration of the folding of the other, our results shed light on a central role for V1 in the determination of the functionality of the chimeras. These results provide a frame for future works aimed at understanding the structural reasons for the interference of genetic diversity in this region with the functionality of the envelope.

## Conclusion

The present study shows that the genetic diversification of the thus far poorly characterized V1 domain is restricted by unsuspected strong functional constraints. Given the extensive diversity of this region in the population and its central role in the immune control of infection, a better understanding of the architectural organization of V1, as well as that of V2, constitutes an important area for understanding the evolutionary trajectories of this region. The perturbation exerted by the variable regions on the structural requirements for retention of Env functionality could define potential new antiviral targets on this protein, crucial for viral infection.

## Methods

### Cells

HEK 293T, 293T CD4^+^CCR5^+^, HeLa-Tat, and TZMbl cells were cultured in Dulbecco's modified Eagle's medium supplemented with 10% fetal calf serum, penicillin (100 UI/ml), and streptomycin (100 μg/ml) (Invitrogen, Carlsbad, NM, USA). The cells were maintained at 37°C with 5% CO_2_.

### Viral sequences and accession numbers

The parental sequences used were primary isolates from subtypes A, B, C, and G. Three isolates from subtype A (GenBank accession numbers AF407156, AF407160 and AF407148), referred to in the present work as A, A1, and A2, respectively; one isolate from subtype B (GenBank accession number: AY835448); four isolates from subtype C (GenBank accession numbers DQ435683, DQ435682, DQ388514, and DQ388515), referred to as C, C1, C2, and C3, respectively; and two isolates from subtype G, G-548 and GenBank accession numbers: AM279346, referred to as G and G1, respectively. All of the viral sequences were provided by the NIH, through the AIDS Research and Reference Reagent Program, Division of AIDS, NIAID, NIH, except the isolates G-548, which was a kind gift from M. Peeters (Institut de Recherche pour le Développement, Montpellier, France) and AM279346 [72], for which the V1V2 region (the region used in the present study) was synthesized chemically by GenScript (Piscataway, NJ, USA).

### Construction of chimeric envelopes

Env chimeras were generated using an overlapping PCR procedure as previously described [54]. Three independent PCR amplifications have been performed to amplify the region to replace (V1V2, C2, and V3), as well as regions located upstream (5′ region) and downstream (3′ region). A subsequent PCR amplification to reconstitute the 5′ region of the gene with the region replaced was performed, and a final PCR amplification will reconstitute the entire chimeric *env* gene. For the final reconstitution, 200 ng for each region of the PCR products was used; these parts contained an overlapping sequence of 30–40 nt. These PCR amplifications were performed using Phusion DNA polymerase (Finnzymes, Espoo, Finland) for 30 cycles. The reconstituted PCR products were gel purified and then cloned into the commercial directional vector pcDNA3.1D-Topo according to the manufacturer’s instructions (Invitrogen, Carlsbad, NM, USA). All of the construct sequences were verified by sequencing (GATC Biotech, Konstanz, Germany), and the expression and processing of all of the chimeras were verified by Western blotting and immunoprecipitation. The swapped domains were defined by the cysteine residues delimiting the V1, V2, and V3 loops. Precisely, the borders of the swapped domains were as follows: 126–196 for V1V2, 126–157 for V1, 157–196 for V2, 196–296 for C2 and 296–331 for V3, where the numbering refers to the amino acids of the gp120 protein of HXB2.

### Entry assay

HEK 293T cells were transfected using the standard calcium phosphate method with the pNL4.3 env^-^ luc^+^ provirus and another plasmid (pcDNA3.1D-Topo-env) carrying the parental or chimeric envelope glycoproteins. pcDNA 3.1D-Topo with no insert was used as a negative control, and a pcDNA3.1D-Topo-env with the envelope from the strain ADA was used as a positive control. Two days post-transfection, the viral particles produced were filtered on 0.45 μM filters, and 50 ng of p24 antigen of each viral preparation was used to transduce 2.5 × 10^5^ HEK 293T CD4^+^ CCR5^+^ cells [54]. All viral solutions were normalized using an enzyme immunoassay for detection of the p24 antigen (Innotest HIV Antigen mAb, Innogenetics, Gent, Belgium). Forty-eight hours after transduction, the medium was removed, cells were washed twice in PBS, lysed, and centrifuged. The supernatant was used to measure luciferase activity according to the manufacturer’s protocol (Promega, Fitchburg, WI, USA) with a Glomax luminometer (Promega, Fitchburg, WI, USA).

### Cell-cell fusion

In this assay, HeLa-Tat cells expressing the Tat protein, which is necessary for the transcriptional activation of HIV-1, were transfected using the calcium phosphate method with a pcDNA3.1 Topo plasmid carrying the envelope protein to test (chimeric, positive, and negative controls mentioned in the entry assay). Two days later, 1.5 × 10^5^ of these cells, expressing envelope proteins at their surface, were co-cultivated in a CO_2_ incubator at 37°C in 12-well plates with 4.5 × 10^5^ cells/well of TZMbl cells containing the luciferase reporter gene under the control of the HIV-1 promoter. After 24 hours of co-cultivation, cells were detached with trypsin and washed, and the same procedure as for the entry assay was followed to lyse and measure luciferase activity.

### Western blot analysis

The expression of wild-type and chimeric proteins was verified by western blotting on the viral particles used for viral entry test and on the cells (expressing the envelopes) used for the cell-cell fusion assay (HeLa-Tat).

For each sample, culture supernatants of transfected HEK 293T cells (containing the viral particles) were filtered through a 0.45 μM filter and viral particles were purified by centrifugation at 130,000 × g for 2 hours through a 20% sucrose cushion. Viral pellets were then solubilized and lysed in RIPA buffer (1× PBS, 1% NP-40, 0.5% sodium deoxycholate, 0.05% SDS). All viral solutions were normalized for p24 antigen content, determined by ELISA (Innotest HIV Antigen mAb, Innogenetics, Gent, Belgium).

HeLa-Tat cells used for the cell-cell fusion assay were washed with 1× concentrated phosphate-buffered saline (PBS) and lysed in RIPA buffer for 15 min on ice. Cell lysates were then centrifuged at 12,000 × g for 20 min at 4°C and the supernatants were recovered for use. Total protein concentrations were normalized for each sample by Bradford assay following the instructions of the Biorad Protein assay (Biorad, CA, USA).

Each sample containing 30 ng of p24 (for western blots on viral particles) and 60 μg of total proteins (for western blots on HeLa-Tat cells) was loaded and separated by electrophoresis in a 4-12% NuPAGE Bis-Tris gel (NuPAGE Novex, Thermo Fisher Scientific, MA, USA). Proteins were then transferred to a polyvinylidene difluoride (PVDF) membrane and blotted with a pool of sera from group M HIV-1 infected individuals (kind gift of J. Mak, Burnet Institute, Melbourne, Australia), followed by horseradish peroxidase (HRP) conjugated sheep polyclonal anti-human IgG (GE Healthcare Life Sciences, Little Chalfont, UK) and also by HRP-conjugated mouse IgG anti human β-actin (Sigma-Aldrich, St Louis, MO, USA) for blots relative to samples of the cell-cell fusion assay. Immunoblots were revealed using a luminol based enhanced chemiluminescence substrate (Pierce ECL Plus western blotting substrate, Thermo Fisher Scientific, MA, USA).

### Comparison of functionality levels between chimeric envelopes

The significance of the differences between the levels of functionality of chimeric envelopes was tested using Student’s *t*-tests by comparing the levels observed for each chimera relative to the level of the wild-type protein providing the backbone. When comparing a chimeric envelope with the wild-type protein that provided the backbone, the levels of functionality of the two proteins being compared were calculated relative to the level of functionality of the reference positive control (HIV-1 ADA Env). The significance of the correlation coefficients was inferred by computing *t* values with the formula t = r/[(1-r^2^)/(N-2)]^1/2^, where r is the regression constant, and N is the number of samples.

### Sequence alignment and estimates of sequence identity

Sequence alignments for the estimate of sequence identity were constructed using MUSCLE 3.8 [55] and manually refined to improve alignment, particularly in regions where the sequence length varied and/or the homology was low, using Mega5 [73]. Sequence identity was calculated pairwise as the fraction of exactly matched nucleotides along the length of each alignment.

## Competing interests

The authors declare that they have no competing interests.

## Authors’ contributions

MH conceived the study and the experimental strategy; carried out all the work described, participated in writing the manuscript. ESL conceived the study and the experimental strategy; participated to the initial experimentation. RG performed the western blotting experiments. MN conceived, designed and directed the study, wrote the manuscript. All authors approved the final manuscript.

## Supplementary Material

Additional file 1: Table S1 Level of functionality of the chimeras with respect to that of both parental wild-type proteins. The values are given as percentage. SD, standard deviation. Click here for file

Additional file 2: Table S2 Sequence similarity of the different structural components of the V1V2 region for the chimeras studied. Results are based on the structure described in reference [31]. The values of sequence similarity have been calculated according to the BLOSUM 62 matrix. The individual regions are defined as in Figure 1A. Click here for file

Additional file 3: Figure S1 Expression of wild-type and chimeric envelope proteins in viral particles. Viral particles were collected 48 h post-transfection and purified on sucrose cushion. Normalized amounts of total viral proteins (based on p24 quantification) were analysed by western blotting using a pool of sera from groupe M HIV-1-infected individuals (kind gift of J. Mak, Burnet Institute, Melbourne, Australia). Panels A, B, C, D, and E correspond to ABA, ACA, AGA, BCB and BGB chimeras, respectively. Positive and negative controls (+ and – signs in the figure) were constituted by viruses containing T-ADA envelope coding plasmid and by viruses obtained after transfection with an empty pcDNA3.1 + pNAL4.3Env^-^ plasmids, respectively. The subtype of the two wild-type proteins used to produce the chimeras and the region replaced are indicated on the top of each panel. Panel F. Western blot of the V1V2/V3 chimeras (see Figure 3A). Parental proteins as well as positive and negative controls are as in panels A-F. In all panels, the bands corresponding to gp120, gp41 and p24 are indicated with arrows. Differences observed between wild-type proteins (A, B, C and G) in the figures could reflect differences either in their level of expression or in the efficiency of their recognition by the sera. Click here for file

Additional file 4: Figure S2 Expression of wild-type and chimeric envelope proteins in HeLa cells. 24 h post-transfection, equalized amount of proteins of HeLa cells lysates (expressing viral envelope proteins) were analysed by western blotting using a pool of sera from group M HIV-1-infected individuals (as for Additional file 3: Figure S1) and by anti β-actin antibody after stripping the membranes. Panels A, B, C, D, and E correspond to ABA, ACA, AGA, BCB and BGB chimeras, respectively. Positive and negative controls (+ and – signs in the figure) were constituted by cells transfected with the T-ADA envelope coding plasmid and by cells transfected with an empty pcDNA3.1 + pNAL4.3Env^-^ plasmids, respectively. The name of each sample is given as for Additional file 3: Figure S1 and the positions of the bands corresponding to the gp120, the gp41, and the β-actin protein is indicated by arrows. Click here for file
