# Supplementary material for: Genetic diversity of the highly variable V1 region interferes with Human Immunodeficiency Virus type 1 envelope functionality
Source: Retrovirology. 2013 Oct 24;10:114. doi: 10.1186/1742-4690-10-114 (PMC3826872; doi:10.1186/1742-4690-10-114)
Supplement: Additional file 1: Table S1 — Level of functionality of the chimeras with respect to that of both parental wild-type proteins. The values are given as percentage. SD, standard deviation. [file 1742-4690-10-114-S1.pdf]

Table S1

| | | wt of reference | Viral entry % | SD (%) |
|---|---|---|---|---|
| V1V2 | ABA | A | 141.5 | 52.1 |
| | | B | 544.4 | 252.5 |
| | ACA | A | 29.1 | 9.2 |
| | | C | 170.6 | 44.7 |
| | AGA | A | 72.5 | 23.0 |
| | | G | 4195.2 | 3673.2 |
| | BCB | B | 1.1 | 1.0 |
| | | C | 2.4 | 2.6 |
| | BGB | B | 3.2 | 3.5 |
| | | G | 65.7 | 60.9 |
| V1 | ABA | A | 44.3 | 13.4 |
| | | B | 151.1 | 81.1 |
| | ACA | A | 15.7 | 10.1 |
| | | C | 134.0 | 75.4 |
| | AGA | A | 9.0 | 1.8 |
| | | G | 830.4 | 788.8 |
| | BCB | B | 9.6 | 4.1 |
| | | C | 14.1 | 7.8 |
| | BGB | B | 0.7 | 0.3 |
| | | G | 17.1 | 9.5 |
| V2 | ABA | A | 66.0 | 25.9 |
| | | B | 260.3 | 145.1 |
| | ACA | A | 53.1 | 8.7 |
| | | C | 359.5 | 185.9 |
| | AGA | A | 125.5 | 38.7 |
| | | G | 11441.9 | 6324.6 |
| | BCB | B | 1.7 | 1.1 |
| | | C | 1.9 | 1.1 |
| | BGB | B | 0.7 | 0.5 |
| | | G | 20.3 | 18.8 |
| V3 | ACA | A | 53.7 | 9.0 |
| | | C | 272.3 | 92.2 |
| | AGA | A | 47.2 | 12.9 |
| | | G | 1.37E+08 | 1.15E+08 |
| | BCB | B | 124.0 | 46.8 |
| | | C | 211.0 | 66.8 |
| | BGB | B | 139.0 | 24.5 |
| | | G | 2907.6 | 1167.0 |
