# Supplementary material for: Genetic diversity of the highly variable V1 region interferes with Human Immunodeficiency Virus type 1 envelope functionality
Source: Retrovirology. 2013 Oct 24;10:114. doi: 10.1186/1742-4690-10-114 (PMC3826872; doi:10.1186/1742-4690-10-114)
Supplement: Additional file 2: Table S2 — Sequence similarity of the different structural components of the V1V2 region for the chimeras studied. Results are based on the structure described in reference [31]. The values of sequence similarity have been calculated according to the BLOSUM 62 matrix. The individual regions are defined as in Figure 1A. [file 1742-4690-10-114-S2.pdf]

chimeras	V1	V2	β-A	V1 loop	β-B	connect	β-C	V2 loop	β-D
A/B	62.5	69.6	100.0	50.0	100.0	66.7	81.8	57.7	100.0
A/C	56.3	78.3	100.0	40.9	77.8	100.0	90.9	66.7	80.0
A/G	52.9	76.1	100.0	33.3	100.0	100.0	90.9	52.4	100.0
B/C	42.5	70.5	100.0	26.7	77.8	66.7	90.9	50.0	80.0
B/G	52.5	77.5	100.0	36.7	100.0	66.7	90.9	53.3	60.0

Table S2
