# Supplementary figures and images for: Genetic diversity of the highly variable V1 region interferes with Human Immunodeficiency Virus type 1 envelope functionality
Source: Retrovirology. 2013 Oct 24;10:114. doi: 10.1186/1742-4690-10-114 (PMC3826872; doi:10.1186/1742-4690-10-114)

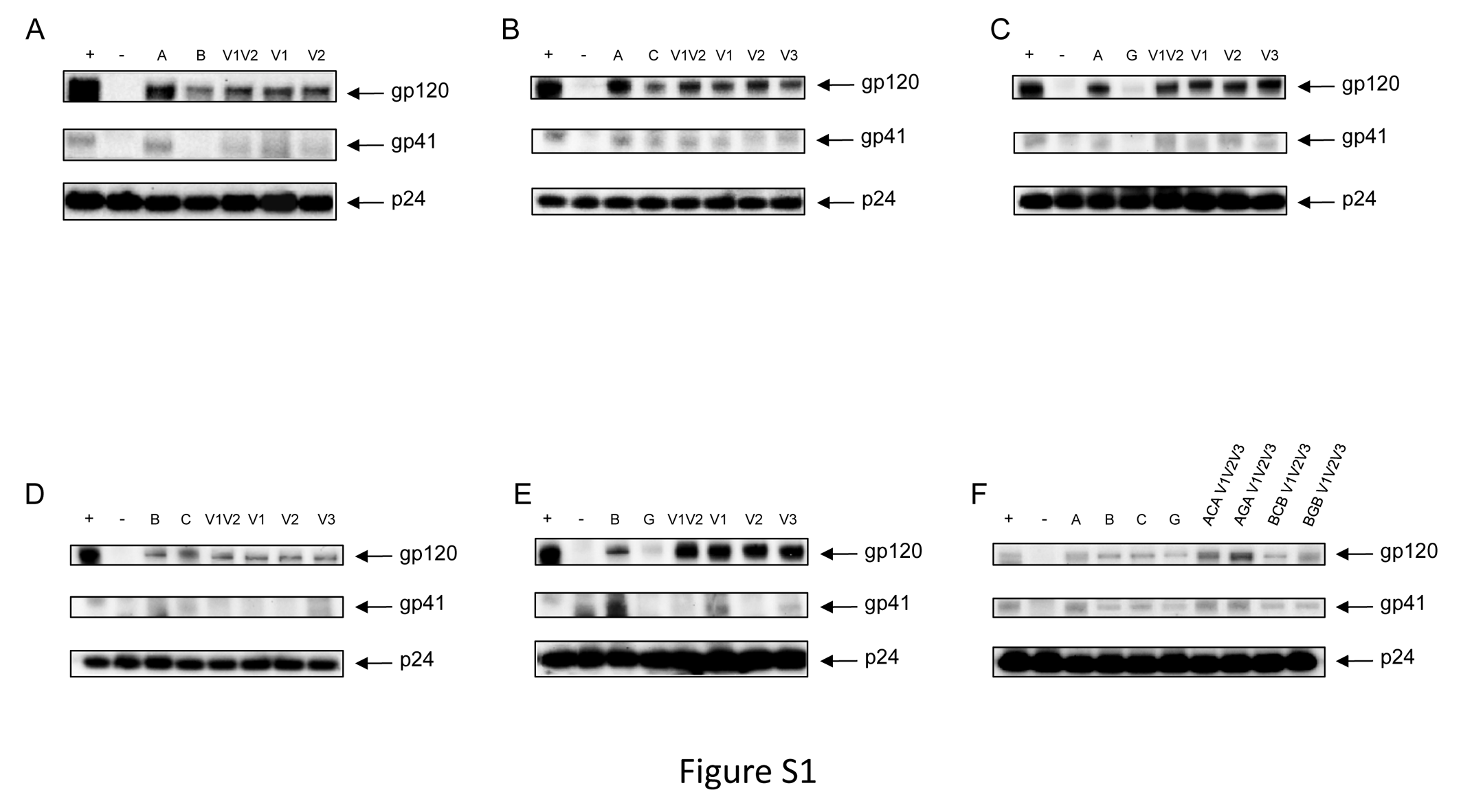

Supplement: Additional file 3: Figure S1 — Expression of wild-type and chimeric envelope proteins in viral particles. Viral particles were collected 48 h post-transfection and purified on sucrose cushion. Normalized amounts of total viral proteins (based on p24 quantification) were analysed by western blotting using a pool of sera from groupe M HIV-1-infected individuals (kind gift of J. Mak, Burnet Institute, Melbourne, Australia). Panels A, B, C, D, and E correspond to ABA, ACA, AGA, BCB and BGB chimeras, respectively. Positive and negative controls (+ and – signs in the figure) were constituted by viruses containing T-ADA envelope coding plasmid and by viruses obtained after transfection with an empty pcDNA3.1 + pNAL4.3Env- plasmids, respectively. The subtype of the two wild-type proteins used to produce the chimeras and the region replaced are indicated on the top of each panel. Panel F. Western blot of the V1V2/V3 chimeras (see Figure 3A). Parental proteins as well as positive and negative controls are as in panels A-F. In all panels, the bands corresponding to gp120, gp41 and p24 are indicated with arrows. Differences observed between wild-type proteins (A, B, C and G) in the figures could reflect differences either in their level of expression or in the efficiency of their recognition by the sera. [file 1742-4690-10-114-S3.tiff]

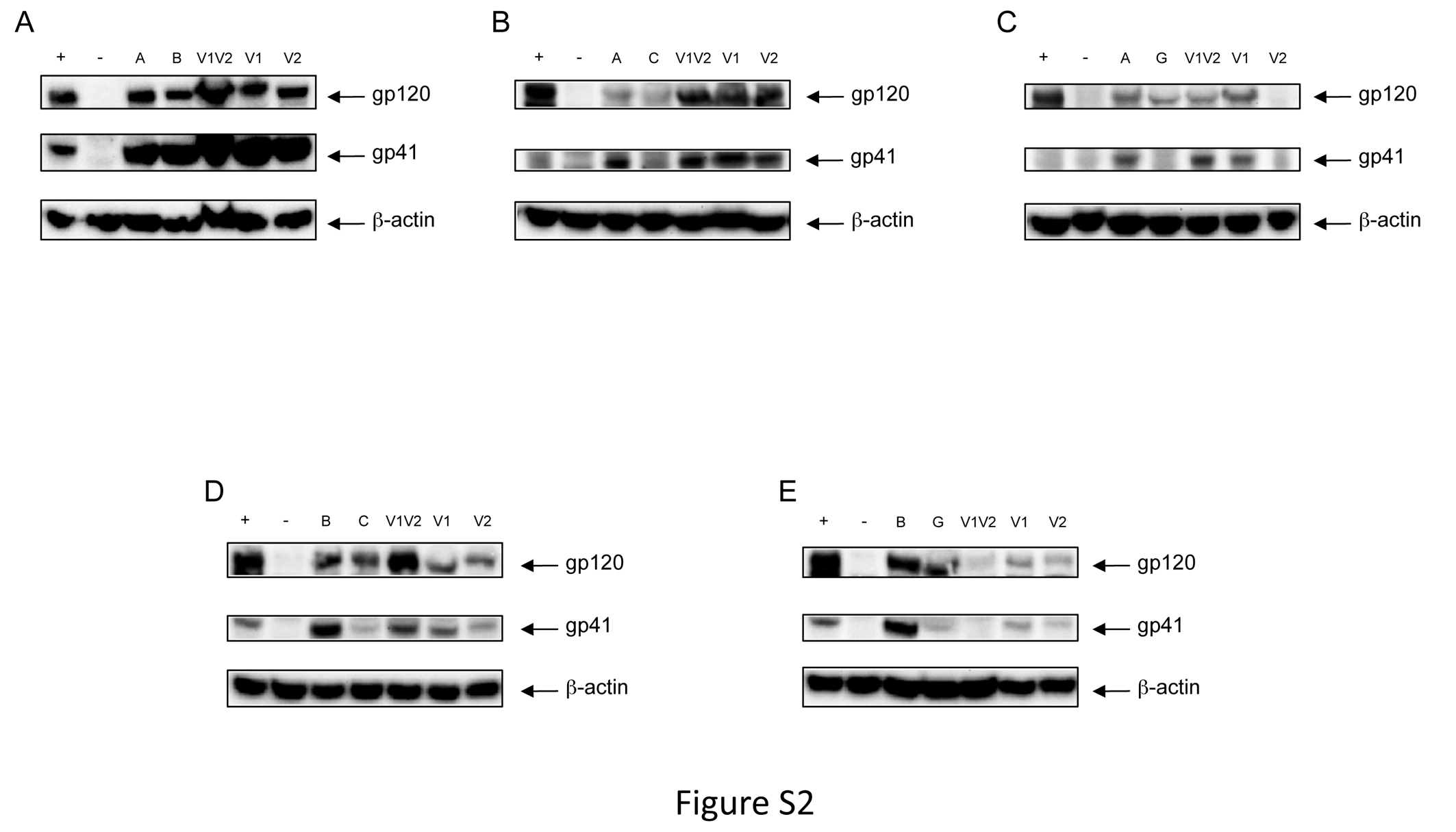

Supplement: Additional file 4: Figure S2 — Expression of wild-type and chimeric envelope proteins in HeLa cells. 24 h post-transfection, equalized amount of proteins of HeLa cells lysates (expressing viral envelope proteins) were analysed by western blotting using a pool of sera from group M HIV-1-infected individuals (as for Additional file 3: Figure S1) and by anti β-actin antibody after stripping the membranes. Panels A, B, C, D, and E correspond to ABA, ACA, AGA, BCB and BGB chimeras, respectively. Positive and negative controls (+ and – signs in the figure) were constituted by cells transfected with the T-ADA envelope coding plasmid and by cells transfected with an empty pcDNA3.1 + pNAL4.3Env- plasmids, respectively. The name of each sample is given as for Additional file 3: Figure S1 and the positions of the bands corresponding to the gp120, the gp41, and the β-actin protein is indicated by arrows. [file 1742-4690-10-114-S4.tiff]
